# Liquid Biopsy: A New Tool for Overcoming CDKi Resistance Mechanisms in Luminal Metastatic Breast Cancer

**DOI:** 10.3390/jpm11050407

**Published:** 2021-05-13

**Authors:** Miriam González-Conde, Celso Yañez-Gómez, Rafael López-López, Clotilde Costa

**Affiliations:** 1 Roche-Chus Joint Unit, Translational Medical Oncology Group, Oncomet, Health Research Institute of Santiago de Compostela (IDIS), Travesía da Choupana s/n, 15706 Santiago de Compostela, Spain; Miriam.gonzalez.conde@sergas.es (M.G.-C.); Celso.yanez.gomez@sergas.es (C.Y.-G.); Rafael.lopez.lopez@sergas.es (R.L.-L.); 2 CIBERONC, Centro de Investigación Biomédica en Red Cáncer, 28029 Madrid, Spain; 3 Translational Medical Oncology Group (Oncomet), Health Research Institute of Santiago de Compostela (IDIS), 15706 Santiago de Compostela, Spain; 4 Medical Oncology Department, University Clinical Hospital of Santiago de Compostela, 15706 Santiago de Compostela, Spain

**Keywords:** breast cancer, CDK inhibitors, liquid biopsy, resistance mechanisms, therapy

## Abstract

Breast cancer (BC) is the most common cancer diagnosed in women worldwide. Approximately 70% of BC patients have the luminal subtype, which expresses hormone receptors (HR+). Adjuvant endocrine treatments are the standard of care for HR+/HER2− BC patients. Over time, approximately 30% of those patients develop endocrine resistance and metastatic disease. Cyclin-dependent kinase inhibitors (CDKi), in combination with an aromatase inhibitor or fulvestrant, have demonstrated superior efficacies in increasing progression-free survival, with a safe toxicity profile, in HR+/HER2− metastatic BC patients. CDKi blocks kinases 4/6, preventing G1/S cell cycle transition. However, not all of the patients respond to CDKi, and those who do respond ultimately develop resistance to the combined therapy. Studies in tumour tissues and cell lines have tried to elucidate the mechanisms that underlie this progression, but there are still no conclusive data. Over the last few years, liquid biopsy has contributed relevant information. Circulating tumour materials are potential prognostic markers for determining patient prognosis in metastatic luminal BC, for monitoring disease, and for treatment selection. This review outlines the different studies performed using liquid biopsy in patients with HR+ metastatic BC treated with CDKi plus endocrine therapy. We mainly focus on those studies that describe the possible resistance mechanisms in circulating tumour-derived material.

## 1. Introduction

Breast cancer (BC) has the highest incidence rate and is the second greatest cause of deaths due to cancer among women worldwide, mainly due to metastasis. The five-year prevalence is nearly eight million worldwide [1]. The luminal subtype represents approximately 70% of the cases and is characterised by the expression of oestrogen and/or progesterone hormone receptors (HR) [2]. Most of the patients diagnosed with primary luminal BC are treated with adjuvant endocrine therapy (ET), such as tamoxifen, anastrozole, letrozole or exemestane, until surgery and/or radiotherapy to block the hormone receptor or to inhibit oestrogen production [3]. However, patients develop endocrine resistance within 2–3 years, on average. In some studies, it was estimated that 30% of patients would develop metastasis, while 6% were in metastatic stages at the time of diagnosis [4,5].

Different mechanisms of endocrine resistance have been identified, such as the upregulation of cyclins, cyclin-dependent kinases (CDKs) and mitogen-signalling pathways (PI3K and RAS); a reduction in CDK-inhibitory proteins (p16, p21 and p27); mutations or the loss of ESR1; and, epigenetic alterations [6]. CDKs act downstream of oestrogen signalling, controlling cell cycle progression. Because these proteins are normally altered in breast cancer, they have been considered to be key target for therapeutic intervention in metastatic settings [5].

The Food and Drug Administration (FDA) and The European Medicines Agency (EMA) approved the combination of cyclin-dependent kinase inhibitors (CDKi) with ET to treat advanced luminal BC. There are currently three cyclin inhibitors on the market (palbociclib, ribociclib, and abemaciclib) that target the ATP-binding domain of CDK4 and 6 [7,8]. Different clinical trials [9,10,11,12,13,14] have demonstrated the benefits of combined CDK4/6 inhibitors plus ET, such as increased progression-free survival (PFS) and survival rates. Nevertheless, not all patients respond to CDKi, and even those who initially respond ultimately progress. There are factors responsible for endocrine resistance that have not yet been identified [15], which complicates the study of resistance to both therapies. The efforts made mainly using primary tumour tissue samples or cell lines have not produced conclusive results regarding the resistance mechanisms, partly due to tumour heterogeneity [7]. Therefore, a paradigm shift is needed for this emergent drug-resistant patient group. Precision oncology through the analysis of liquid biopsies has emerged as an attractive opportunity for this. Contrary to that in classical oncology, the therapeutic strategy in precision medicine is based on the distinctive molecular characteristics of patients. Thus, the objective is to tailor patient therapy by studying biomarker profiles while reducing the harmful effects on healthy cells. BC treatment is a clear example, where the treatment selection depends on the subtype [16].

In recent years, liquid biopsy has become a tool for elucidating tumour evolution in real time and guiding systemic treatment selection for precision medicine. Moreover, it provides information on the tumour’s genomic profile and burden, without invasive procedures. In addition, they can be performed longitudinally whenever needed. Although its analytical and clinical validity is evident, clinical trials that incorporate the analysis of tumour-derived materials, such as circulating tumour DNA (ctDNA) or circulating tumour cells (CTCs), are necessary for clinical decision making. For instance, the detection of *PIK3CA* mutations in ctDNA to guide treatment selection is an example of a clinically useful ctDNA assay.

The literature was reviewed to evaluate the use of liquid biopsy, for the analysis of tumour-derived material, in order to identify predictive biomarkers in HR+/HER2− metastatic BC (mBC) patients that were treated with CDK4/6 inhibitors plus endocrine therapy.

## 2. Inhibition of Cyclin-Dependent Kinases 4/6 (CDK4/6) in Combination with Endocrine Therapy for HR+/HER2− Metastatic Breast Cancer

**Figure 1:**
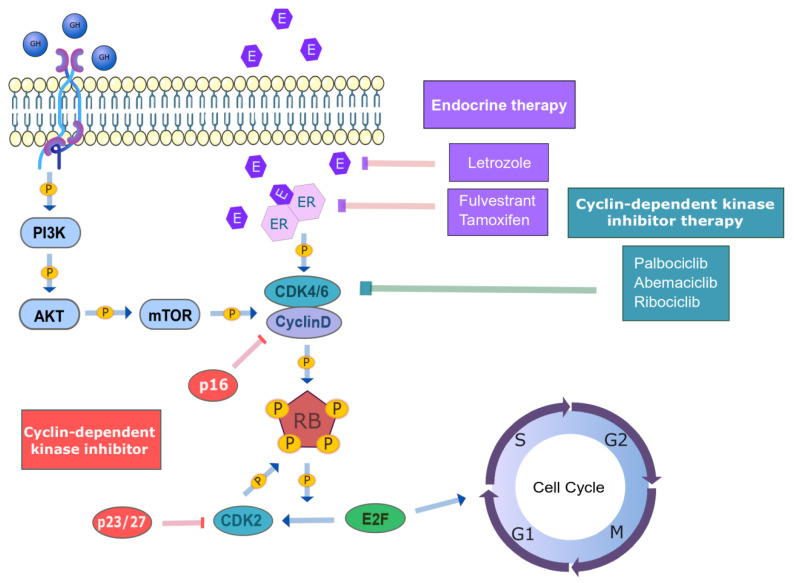
Regulation of the cell cycle in HR+/HER2- mBC patients. The regulation of the cell cycle is mediated by the CCND1–CDK4/6-RB axis. The CCND1–CDK4/6 complex phosphorylates the RB protein, which releases E2F transcription factors. The latter lead to the G1/S transition of the cell cycle. The cyclin–CDK complexes are, in turn, regulated by other cyclins or intrinsic CDK inhibitors (INK4 and CIP/KIP family members) (in red). The current treatments in HR + mBC are endocrine therapy (in purple) and CDK inhibitors (in green). (HR: hormone receptor, HER2: human epithelial growth factor receptor 2, CCND1: cyclin D1, CDK4/6: cyclin-dependent kinase 4/6, RB: retinoblastoma, E2F: E2F transcription factor, CDK inhibitors: cyclin-dependent kinase inhibitors, INK4: inhibitors of CDK4, CIP/KIP: CDK interacting protein/kinase inhibitory protein).

Cell cycle progression is regulated by cyclin-dependent kinases and cyclins. It has been described that the CCND1–CDK4/6 complex controls the G1/S transition [8,17], which is normally upregulated in HR+/HER2− BC. Thus, *CCND1* (29% in luminal A and B) and *CDK4* (14% in luminal A and 25% in luminal B) are commonly amplified. The CCND1–CDK4/6 complex phosphorylates the retinoblastoma protein (pRB), a negative regulator of cell cycle progression. The inactivation of RB releases E2F transcription factors, which activate the transcription of genes that are implicated in DNA replication and cell cycle progression [3,8,18,19] (Figure 1).

Pharmaceutical companies have designed treatments to inhibit CDK4/6 to arrest the cell cycle at G1. The first generation of CDKi was nonspecific, of limited efficacy and affinity, and considerably toxic [5,8,20]. Computer-aided drug design is being used to develop CDKi with better potency, selectivity, and pharmacological properties, and the spatial structure and inhibition activity of CDKs are also being studied [21,22]. Palbociclib and ribociclib have more than 100-fold-higher affinities for CDK4/6 than other CDKs, while abemaciclib has only an approximately six-fold higher affinity. A more profound understanding of molecular differences is necessary for the precise use of this drugs in clinical settings, although the comparable efficacy of these inhibitors was confirmed by an increase in the PFS, independent of the patients’ features [23]. 

It was described that, in mBC patients that were previously treated with two or more hormonal treatments, CDKi resulted in a higher rate of clinical benefit and PFS than in those patients treated with one hormonal therapy or none. It was also observed that the therapeutic response was independent of the nuclear expression of *RB1*, Ki-67 index, p16 loss, or *CCND1* amplification in the tumour tissue. Because of this synergetic effect, several clinical trials were carried out to determine the efficacy of the combined therapy as a first-line treatment in mBC patients [24,25].

The PALOMA clinical trials (1, 2, and 3) assessed the safety and tolerability of palbociclib plus letrozole or fulvestrant as a first-line therapy in HR+/HER2- mBC patients with or without prior treatments. As in previous preclinical studies, a higher clinical benefit rate and PFS were demonstrated in patients that were treated with the combined therapy than with ET alone or plus placebo [8,10,13,26,27]. It was also demonstrated that a *CCND1* amplification and/or loss of p16 in the primary tumour did not improve the efficacy of therapy [26].

The MONALEESA trials (2 and 3) assessed the safety and toxicity of ribocilib plus letrozol or fulvestrant in HR+ and HER2− mBC patients. The results reported an improved PFS and manageable toxicities with the combined therapy than with ET plus placebo [8,28,29]. In MONALEESA-7, triplet therapy with ribociclib, goserelin, and tamoxifen or aromatase inhibitors (AI) were approved due to resulting in a higher PFS and overall survival than those that were patients treated with placebo, goreselin, and tamoxifen [28,30].

The MONARCH trials (1, 2, and 3) determined the activity of abemaciclib as a single agent or in combination with fulvestrant or a non-steroidal AI in HR+/HER2− mBC patients as first- or second-line therapies. The combination significantly improved the PFS and overall survival [31,32,33,34].

In summary, CDKi plus ET increases the life span of HR+/HER2− mBC patients, but these patients eventually develop resistance. Preclinical evidence suggests that different cell cycle regulators and oncogenic drivers may be involved in CDKi resistance. First, preclinical cell line studies have revealed some candidate resistance mechanisms, such as the upregulation of the Pi3K/AKT/mTOR pathway; the loss of *RB1*; acquired mutations in RB1 inhibitors; the amplification or mutation of *FGFR*; the upregulation of *PDK1, MYC* or *SKYPE*; and, the overexpression of CDK4/6. Likewise, the formation of CCNE–CDK2 and CCND1–CDK2 complexes can control the cell cycle progression after CCND1–CDK4/6 inhibition [19]. Secondly, Wander et al. identified eight possible resistance mechanisms in patients that were resistant to CDKi, and they confirmed the results in cell lines resistant to this therapy: *RB1* allelic disruption; amplifications and/or mutations in *AKT, RAS, AURKA, CCNE2, FGFR2,* and *ERBB2*; and, the loss of *ESR1* [35]. It is necessary to determine whether these mechanisms are clinically relevant in treated HR+/HER2− mBC patients. 

## 3. Liquid Biopsy as an Innovative Tool for Deciphering Resistance Mechanisms 

**Figure 2:**
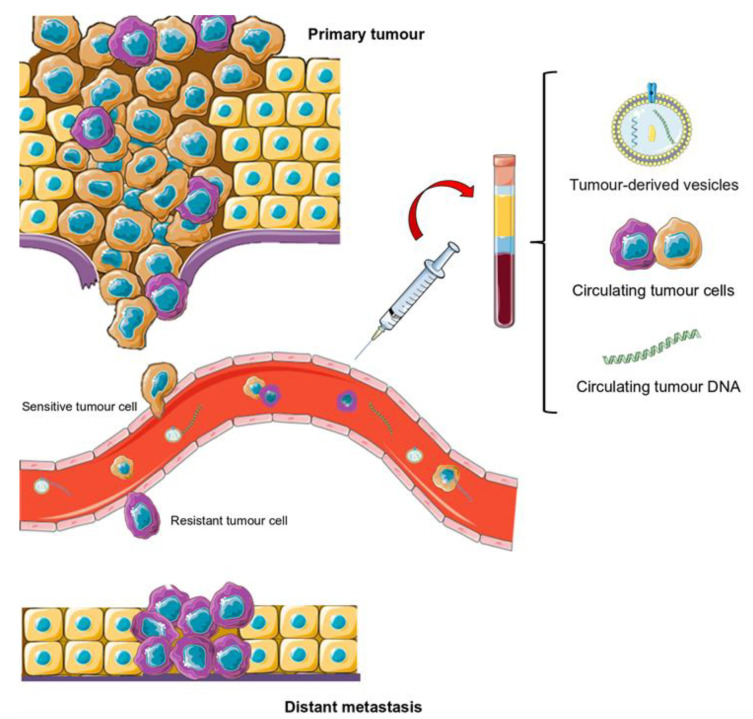
Scheme of metastatic progression and potential use of liquid biopsy. In the bloodstream, there are different circulating tumour cell (CTC) subpopulations. Those that have survived the therapy and become resistant are responsible for the recurrence and progression of the disease to distal locations. Liquid biopsy permits the sampling of these and other tumour entities, such as circulating tumour DNA (ctDNA) or extracellular vesicles. As such, serial liquid biopsy throughout therapy is useful for studying the appearance of treatment resistance.

Tumours are heterogeneous and dynamic units that evolve throughout the disease, sometimes due to the selective pressure that is exerted by the different treatments received [36]. Despite the fact that primary tumour biopsies have been extensively utilised, this technique has multiple downsides: invasiveness, no representation of the tumour’s genetic landscape, and an inability to facilitate serial testing. Therefore, primary biopsy data may not provide real information on the current molecular characteristics of a given tumour [36,37]. However, metastatic tissue biopsies are not always feasible, due to inaccessible tumour sites or the impossibility of sampling multiple metastatic sites, and they do not represent tumour heterogeneity. In the last decade, liquid biopsy overcomes tissue biopsy limitations through the study of tumour-derived material from biological fluids (blood, urine, saliva, etc.). Thus, the main studied tumour entities are circulating tumour cells (CTCs), circulating tumour DNA (ctDNA), and tumour-derived extracellular vesicles (EVs) due to their diagnostic and/or prognostic potential (Figure 2) [38]. Recently, circulating tumour-derived proteins, circulating tumour RNA, and tumour-bearing platelets have also been described as potentially relevant markers [38]. These tumour entities allow for the assessment of tumour heterogeneity, allow the tracking of a tumour’s genomic evolution during treatment, and provide information regarding the biology behind the metastatic development [39,40]. Therefore, the longitudinal sampling of circulating tumour material may help oncologists to predict disease progression and treatment failure, and tailor patient therapy [41].

## 4. Circulating Tumour DNA (ctDNA) Analysis as a Tumour Biomarker in HR+/HER2− mBC Patients

The mechanisms of ctDNA release are not well described, but two release processes are accepted: (a) an active mechanism due to the necrosis or apoptosis of tumour cells; and, (b) a mechanism of active release by the tumour itself, which could constitute a system of communication with the environment. Thus, the amount of ctDNA depends not only on the number of dead cells, but also on the metabolism of the tumour, tumour location, vascularisation, rate of proliferation, etc. [42,43,44]. ctDNA analysis is a minimally invasive approach for diagnosis, as well as for detecting residual tumours and metastases, but mainly for identifying resistance mutations at clinical progression, permitting therapy selection [43,44]. For ctDNA analysis, Next-generation sequencing (NGS) and droplet-based digital PCR (ddPCR) are the main techniques used (Table 1). NGS is a multiplex technology that can be used to detect a large number of (novel) genetic alterations covering complex genomes [45]. On the contrary, ddPCR and BEAMing assays are rapid, sensitive, and precise with little input material, but the alterations must be known in advance [46,47].

In recent years, ctDNA has been used as a promising approach to identify resistance to CDKi plus ET in HR+/HER2− mBC patients (Table 1). In primary tumours, an association between biomarkers and therapy responses was not observed [24,26,48]; however, in ctDNA, some therapy-related alterations were identified, as described below. 

O’Leary et al. detected subclonal *RB1* mutations in nine out of 195 (5%) patients that were treated with palbociclib or ribociclib plus ET at the end of treatment. The clinical prevalence of *RB* mutations in primary BC tumours is low, while their prevalence in patients resistant to *CDK4/6* inhibitors with prior endocrine therapy is unknown [3]. As *RB* alterations were detected in ctDNA after exposure to CDKi, it was assumed that they were the result of selective pressure from the therapy. However, as these mutations were part of a subclonal population, their analysis in tumour-derived material was complex [3,58]. Furthermore, *RB1* mutations were only selected in *ESR1* wild-type tumours, but not in those with fulvestrant resistance, due to ESR1 mutations, which suggested that several resistance pathways were involved [58].

There is some controversy concerning *ESR1* subclonal mutations. Some of the studies have detected *ESR1* mutations in patients resistant to combined therapy [35], while other patients were sensitive to CDKi, regardless of *ESR1* status [49,50]. A reduction in ESR1 ctDNA abundance was also observed after two weeks of therapy, but did not improve the PFS or predict the sensitivity [51]. Studies focusing on ctDNA analysis found that substantial *ESR1* loss and gain alterations reflected therapy pressure in different subclones [3]. Likewise, it seems that patients with *ESR1* mutations at baseline exhibited worse PFS than those with wild-type mutations due to therapy pressure [13]. Studies that were carried out after the completion of treatment suggested that the loss of the *ESR1* mutation was more common in patients treated with palbociclib plus fulvestrant therapy than in those receiving the placebo, but this loss did not improve the PFS or predict the sensitivity [51]. Nevertheless, further analyses are required to understand the role of *ESR1* mutations in resistance to polytherapy. A PADA-1 trial (phase III) assessed *ESR1* mutations in ctDNA to evaluate the efficacy and safety of switching the ET (from AI to fulvestrant) combined with palbociclib. Likewise, *ESR1* mutations (E380, L536, Y537, and D538 hotspots) were monitored by ddPCR [59]. The preliminary results show that *ESR1* mutations were uncommon in patients that were not treated with AI in the neoadjuvant setting. In addition, a one-month treatment with palbociclib and AI decreased the *ESR1* mutation rate [60,61].

Other proposed resistance mechanisms imply PI3K alterations. The PI3K gene is described as a gene with a strong pattern of variant acquisition and a loss of clones during treatment [3,48]. There was no association between PI3K alterations and PFS, the benefit of the combinatorial therapy or the HR status [48]. However, it was observed that a reduction in the *PIK3CA* ctDNA level after two weeks of treatment predicted the long-term clinical outcome (four vs. 11 months) [51].

ctDNA *FGFR1* alterations have also been linked to patients’ outcomes. Formisano et al. observed that, at baseline, 20 out of 247 (5%) patients possessed an alteration in *FGFR1*, which was associated with worse outcomes. Of those patients that progressed to poly-therapeutic treatment, 41% (14/34) possessed an *FGFR1* alteration, which suggested a connection between *FGFR1* mutations and progression [52]. In addition, the analysis of the *FGFR1* mRNA expression in tumour samples showed that patients with high *FGFR1* mRNA expression exhibited a worse PFS when treated with letrozole plus ribociclib. It was also identified that the mRNA overexpression and amplification of *FGFR1* reduced the sensitivity to palbociclib and fulvestrant treatment in vitro [52]. Other studies observed that alterations at baseline in *PIK3CA, TP53, CDH1, FGFR1,* cell cycle-related genes, or genes involved in receptor tyrosine kinase signalling did not predict the response to ribociclib plus letrozole therapy [53]. However, Neven et al. found that, regardless of the ctDNA gene alteration status, the PFS was higher in patients that were treated with ribociclib [61].

## 5. Circulating Tumour Cell (CTC) Analysis as a Novel Biomarker for Managing HR+/HER2− mBC Patients

Cancer heterogeneity results in tumour cell subpopulations with different genomics, rates of proliferation, aggressiveness, and drug sensitivities. These cancer tumour cells are released into the blood circulation actively, via epithelial–mesenchymal transition, or passively, detached from the primary tumour or metastasis as single cells or clusters, which have greater metastatic potential. Thus, the presence of ≥5 CTCs per 7.5 mL of blood was associated with poor outcomes in metastatic breast and prostate cancer, while ≥3 CTCs per 7.5 mL of blood was so in colorectal cancer patients [42]. In early breast cancer, CTC count is also a prognostic factor for patients treated by neoadjuvant chemotherapy [62] and after two years of chemotherapy [63]. Regarding the correlation of CTC enumeration with patients’ TNM staging, Camara and colleagues found that the number of CTCs correlated with tumour size [64]. This result is in agreement with other studies where it was observed a correlation between the reduction of CTC counts and a decrease in tumour size after chemotherapy [65]. A moderate association between detection of ≥1 CTC and positive lymph node involvement has also been reported in neoadjuvant or adjuvant patients [62]. However, other groups found no association between CTC counts and TNM stage or lymph node involvement [66]. These discrepancies may be due to the different methodologies used. Therefore, the results are not comparable. The CellSearch^®^ system (Menarini Silicon Biosystems, Bologna, Italy) is the only platform validated by the FDA for CTC enumeration. It is an immunomagnetic method that uses the epithelial antibody EpCAM to positively enrich CTCs. Next, it is performed a staining for cytokeratins 8, 18, and/or 19; CD45; and, nuclei for CTC enumeration [42]. However, it ignores CTC subpopulations with mesenchymal or stemness phenotypes [42]. Despite the technological advances, the low number of CTCs in the blood is still a hindrance to their isolation and characterisation [67]. Thus, sampling higher volumes of blood by leukapheresis is an alternative being explored [42,68,69]. Likewise, studies at the single-cell level have been of great relevance in unravelling the tumour heterogeneity in BC and other types of tumours, studying resistant clones, and determining the resistance mechanisms and therapeutic responses [42,70]. Thus, a study conducted by De Luca et al., carried out in a patient with breast cancer, observed that most of the CTC mutations that were detected at the beginning of the study disappeared during treatment, while new mutations emerged [71]. Furthermore, the study of CTCs has already been shown to be useful for deciphering resistance mechanisms in luminal patients treated with endocrine therapy [72,73].

Regarding HR+/HER2− mBC patients treated with combinatorial CDKi and ET therapy, Galardi et al. have recently analysed CTCs to identify the response to palbociclib. CTC enumeration, which was performed by CellSearch System, did not predict PFS at baseline or the clinical benefit at any time point. After one cycle of therapy, it was found that samples with ≥1 CTC had a worse PFS. Besides, those patients with ≥5 CTC develop resistance earlier. RB expression was also analysed but there was no correlation with clinical benefit [55]. Although this is the first study reported based on CTCs enumeration or molecular analysis in these patients, a bigger cohort and more analysis are required, as CTC characterisation will increase their clinical utility. Interestingly, in 2020, Koch et al. established an ER+ breast CTC line that was derived from a patient with luminal metastatic breast cancer. The cell line was demonstrated to be genetically identical to the original CTCs, a case that had never been described before. In addition, it was observed that palbociclib reduced the cell line growth, even at low doses in this novel CTC line [67].

As future perspectives, the study of CTCs will allow omics analysis (gene expression, proteins, metabolites, etc.), as well as functional tests in vitro and *in vivo*, in order to elucidate the metastatic process and the underlying resistance mechanisms [74,75,76]. Table 2 describes the main advantages of studying CTCs compared to other circulating tumour entities.

## 6. Analysis of Extracellular Vesicles (EVs), a Possible Biomarker of CDKi

EVs play an important role in the communication between cells in both healthy tissues and tumour microenvironments. They can be generated within endosomes, forming smaller EVs of 50–100 nm in diameter, called exosomes, or through budding directly from the plasma membrane, resulting in microvesicles that can vary from 50 nm to 10 μm in diameter [80]. The EVs contain a wide variety of biomolecules, including RNA, lipids, proteins, and DNA [81]. They allow cancer cells to establish crosstalk between the tumour and the stroma, and to take part in processes, such as tumorigenesis, angiogenesis, invasion, and metastasis [81]. Likewise, it was described that they can transmit drug resistance through functional proteins and microRNAs (miRNAs) [82].

Del Re et al. identified miRNAs from exosomes in plasma from mBC patients that were treated with CDKi plus ET. These patients, with elevated levels of *CDK4* expression, had prolonged PFS and better therapeutic responses. In addition, increases in *CDK9* and *TK1* mRNA copies were related to clinical resistance [56]. These preliminary results established exosomes as a promising biomarker for monitoring the outcomes of CDKi therapy [56]. Another recent study demonstrated a new exosome-mediated mechanism of resistance to CDKi that was acquired through extracellular signalling, involving exosomal miRNA [57]. Increases in CDK6 protein and mRNA expression were observed in palbociclib-resistant BC cell lines. CDK6 knockdown re-sensitised the cells to palbociclib treatment, which indicated this cyclin as a key mediator of the resistance mechanism. Through co-culture experiments, it was found that palbociclib-sensitive cell lines could acquire resistance to the drug when co-cultured with resistant cells or with their exosomes, suggesting that the resistance could be transmitted through extracellular vesicles. EV analysis in these cell lines identified the miRNA miR-432-5p as a possible mediator of CDKi resistance [57]. The expression of miR-432-5p was found to be 1.8-fold higher in biopsies from luminal BC patients with intrinsic or acquired CDKi resistance than in those from patients with sensitive tumours. A 2.7-fold decrease in *SMAD4* expression was also observed in resistant tumours, which indicated a TGF-β pathway suppression mediated by miR-432-5p, as it interacts with numerous genes from the TGF-β pathway. Using both in vitro and in vivo models, the authors provided evidence of the loss of acquired resistance following drug removal, suggesting that CDKi could be used again after adequate drug breaks [57].

## 7. Conclusions

Liquid biopsy is a fundamental tool for studying tumour heterogeneity, the main cause of therapeutic failure in cancer patients. Therefore, changes in the molecular profiles of primary tumours and metastasis can be longitudinally studied via a non-invasive and real-time approach [53]. Various trials have demonstrated the benefits of combined CDK4/6 inhibitors plus endocrine therapy in HR+/HER2− mBC, such as increasing the PFS, regardless of menopausal status, prior therapies, endocrine sensitivity, and the site of metastasis [23,60]. However, certain limitations remain to be resolved, such as the lack of predictive biomarkers with which to select patients or detect resistance [24]; these are among the current topics in the context of luminal metastatic breast cancer.

In this regard, studies that were carried out on ctDNA point out that mutations acquired at the end of treatment were related to a longer PFS in patients who progressed to polytherapeutic treatment. It is likely that tumours that progress early do not acquire mutations, due to the lack of treatment pressure. Therefore, other resistance mechanisms can affect early progression, so it is important to consider the intrinsic resistance when selecting the next line of treatment. O’Leary et al. described that resistance to fulvestrant boosts resistance to combinatorial therapy, mainly in tumours that could progress during CDKi treatment with active ER signalling. One possible explanation is that tumours can adapt to CDK4/6 inhibitors if ESR1 signalling is not correctly suppressed, but, when considering the lack of consensus regarding *ESR1* status and CDKi sensitivity, further studies are needed. However, this suggests that ET could be a resistance driver [3]. Other proposed biomarkers, such as *PI3K*, indicate that a reduction in the mutated fraction extends the time to progression, while the data for *FGFR* are contradictory, which prevents reliable conclusions from being drawn. Despite the significant potential of CTCs, more studies on CTC gene expression analysis and molecular characterisation in a metastatic clinical setting have to be done. Deciphering changes in expression after combined therapy, especially in patients with intrinsic resistance, may be a milestone that allows the interpretation of the underlying resistance mechanisms (González-Conde et al., unpublished data). This is of particular interest, as CTCs that survive therapy can colonise distal organs and contribute to disease progression. Likewise, CTC lines are functional models with which to test drug activity and decipher intrinsic mechanisms that are involved in the metastatic cascade. Finally, EVs are being considered as novel biomarkers for determining the therapeutic response and identifying resistance mechanisms, but it is necessary to continue with these investigations. In summary, for data validation, the study of tumour-derived material via a comprehensive approach could be of great interest due to ctDNA, CTCs, and EVs providing complementary information.

Concerning combined therapy, one limitation is the lack of knowledge regarding the contribution of each treatment or if the resistance is due to the action of both drugs. Furthermore, in the clinical context, we must assess whether the mutational state prior to treatment determines the therapeutic efficacy. In addition, patients with intrinsic resistance should be studied to detect novel resistance mechanisms, as the evolution of driver gene mutations is infrequent due to the lack of selective pressure.

Simple models of genetically encoded sensitivity do not reflect patients’ genetic landscape, owing to the genetic complexity of cancer and possible mechanisms of acquired resistance [5]. Knowing the profile of each patient at a given point in time will allow the selection of the most beneficial therapeutic sequences. Thus, the future clinical outlook should be based on the molecular characterisation of primary tumours and metastasis, as well as tumour-derived material (ctDNA, CTC or EVs) at different time points in the metastatic clinical setting. The holistic liquid biopsy analysis of tumour material will change the current clinical paradigm for luminal BC patients, in such a manner that the best treatments will be selected and resistance will be overcome. Several clinical trials consider ctDNA to be a major informative biomarker, but other circulating tumoral entities that could provide transcriptomic data that are related to the metastatic cascade or resistance acquisition have not yet been accounted for. The clinical implementation of liquid biopsy is underway and, despite current technological limitations, it only is a matter of time before their use becomes universal [24,42,44].

## Figures and Tables

**Table 1:** Summary of the literature review based on the analysis of tumour-derived material from HR+/HER2- advanced breast cancer patients using liquid biopsy. (NGS: Next Generation Sequencing, ddPCR: droplet-digital PCR, qPCR: quantitative PCR)

Tumour-Derived Material	Biomarker	Therapy	Technique	References
**ctDNA**	*RB* mutations	PALOMA 3: Palbociclib plus fulvestrant	Exome sequencing NGS ddPCR	O’Leary B, Cutts RJ, Liu Y, et al. [3]
		Palbociclib plus fulvestrant Ribociclib plus letrozole	NGS	Condorelli R, Spring L, O’Shaughnessy J, et al. [49]
	*ESR1* mutations	PALOMA 3: Palbociclib plus fulvestrant	Exome sequencing NGS ddPCR	O’Leary B, Cutts RJ, Liu Y, et al. [3]
			ddPCR	Fribbens C, O’Leary B, Kilburn L, et al. [50]. O’Leary B, Hrebien S, Morden JP, et al. [51]
		Palbociclib plus AI/fulvestrant	Exome sequencing NGS	Wander SA, Cohen O, Gong X, et al. [35]
	*FGFR1* mutations	MONALEESA-2: Ribociclib plus letrozole	NGS qPCR	Formisano L, Lu Y, Servetto A, et al. [52]
			NGS	Hortobagyi GN, Stemmer SM, Burris HA, et al. [53] Neven P, Petrakova K, Bianchi GV, et al. [54]
	*PI3K* mutations	PALOMA 3: Palbociclib plus fulvestrant	Exome sequencing NGS ddPCR	O’Leary B, Cutts RJ, Liu Y, et al. [3]
			BEAMing assay ddPCR	Cristofanilli M, Turner NC, Bondarenko I, et al. [48] O’Leary B, Hrebien S, Morden JP, et al. [51]
**CTC**	CTC enumeration RB1 expression	TREnd trial: Palbociclib monotherapy Palbociclib plus ET	CellSearch System ddPCR	Galardi F, De Luca F, Biagioni C, et al. [55]
**EVs**	CDK9/4 and TK1 mRNA copies	ECLIPS study: CDKi plus endoncrine theraphy	ddPCR	Del Re M, Bertolini I, Crucitta S, et al. [56]
	miRNA 432-5-p	Palbociclib plus letrozole/fulvestrant	mRNA/miRNA array NGS	Cornell L, Wander SA, Visal T, Wagle N, Shapiro GI [57]

**Table 2:** Summary of the advantages and disadvantages of different circulating tumour entities (ctDNA, CTCs, EVs). (ctDNA: circulating tumour DNA, CTC: circulating tumour cells, EVs: extracellular vesicles).

	CTC	ctDNA	EVs
High concentration	NO	NO	YES
Study of tumour mutations and methylation patterns	YES	YES	YES
Study of tumour RNA transcription profile	YES	NO	YES
Detection of systemic changes and inflammation	NO	NO	YES
Use of biobanked samples	YES *	YES	YES
Functional assays	YES	NO	YES
Morphological characterisation	YES	NO	NO
Validated predictive value	YES	NO	NO

* Biobanked samples do not allow performing functional analysis [77,78,79].
